# Surgical Cases

**Published:** 1881-11

**Authors:** Herman Mynter


### Surgical Cases.

Reported by Herman Mynter, M. D.

July 7, 1881, Mr. B., a strong and healthy butcher, injured his right knee-joint with an axe in chopping meat. He received a wound about one inch long, on the inner side of the joint, about one inch from the margin of the patella. The family physician examined the wound, and seeing a free discharge of synovia, and recognizing the importance of the lesion, enjoined absolute rest and hermetically sealed up the wound. The patient, who had once before had a similar wound, which had healed without giving any trouble, discarded the advice of his physician and kept about his business for four days, until the increasing swelling and pain made it impossible for him to do so.

July 21, 14 days after the accident, I saw the patient, and took charge of him at the request of the family physician. He had then considerable fever, the temperature being 103, and complained of great pain, restlessness and lack of appetite. The right knee, which was considerably bent, was enormously swollen and expanded by intracapsular exudation.

On the inner side was seen a widely gaping ulceration with flabby, pale, unhealthy granulations. By examining the joint and slightly compressing it, a stream of light-yellow fluid with large fibrinous deposits spurted out three or four inches, and continued to do so till the joint was empty. A large canula was introduced, and the joint syringed out with 2 per cent. carbolized water. The wound was dusted with pulverized iodoform and covered with lint with an ointment of boracic acid and cosmoline (1 to 8). Posterior splint, compression with Martin's elastic bandage and ice bladders were applied. The fever disappeared in a few days, but the exudation returned in the joint, and during the following weeks continued to empty itself through the wound as soon as a moderate amount of fluid had collected in the joint. The wound gradually diminished in size, and at the end of the three weeks only a small fistula was left, through which synovia continued to flow. The fistula kept open three weeks longer in spite of cauterization and compression, but was at last healed by continually tapping the joint, whenever the fluid gathered, while a continual compression of the joint and fistula was used. As soon as the fistula had healed, the patient was put under the influence of ether, and the joint, which had become perfectly stiff, freely moved and the adhesions broken up.

Massage and frictions were practiced daily for a week, and the patient then encouraged to walk with crutches. A diffuse swelling of the lower third of the femur of periosteal nature delayed his recovery for some weeks, but he is now able to walk without crutches and the motion of the joint, although still somewhat impaired, is gradually getting better and better.

---

In the BUFFALO MEDICAL JOURNAL 1879 to '80 (vol. 19, page 393), appeared a translation of an article by Dr. Volkman about puncture of hæmartros, in which two points were brought out, 1st, that in cases of traumatic hydarthrosis the blood remains perfectly, or almost perfectly, fluid for some time and may be removed by puncture. He found it always fluid during the first three days; and once after six days, and three times after eight days, while in a large number of other cases, punctured between the fourth and eight days, the principal part of the blood was fluid, but some coagula were removed through the trocar, and others left behind; 2d, that anchylosis with total obliteration of the joint may occur in cases, in which the coagulated blood is quickly organized, which may happen, if uniform layers of clot are deposited on the inner surface of the synovial membrane and the secretion of synovia suppressed perfectly by it. Both points are of great importance, especially, I should think, in cases of fracture of the patella. Every surgeon has seen, how easy it is to bring both fragments together immediately after the injury, and how difficult it is, 24 hours afterwards, on account of the large accumulation of blood in the joint. And when this blood at last is absorbed, the muscles have contracted so much that we are glad if we can get a short ligamentous union. If we relieve the joint of the accumulated blood by aspiration, I should, from theoretical reasons, consider it much more easy to obtain a short fibrous or even a bony union. The first point, about the fluidity of the blood, I have had opportunity to verify lately. The patient, a little boy, five years of age, fell on the street and hurt his right knee. I saw him three days after the accident, in consultation with Dr. Wetzel, and found the joint greatly swollen from intracapsular bleeding, and the limb bent and fixed at an acute angle. Under chloroform the limb was straightened and the joint aspirated and about 50 grams of dark fluid blood removed, which contained no coagula. A posterior splint, Martin's elastic bandage and ice bladder were applied, and in about eight days the little fellow was about the street again with a perfect joint.

---

August 8th, 1881, I was called to Suspension Bridge by Dr. Talbot to operate for strangulated hernia. The history of the case, from notes of Dr. Talbot, is as follows:

The patient, Mr. S., 30 years of age, developed a rupture fourteen years ago in Germany, and for three weeks the rupture had remained out and resisted all attempts of reduction. At last it returned to its place of its own accord. Five years ago it appeared again, and was reduced by a surgeon after two hours' manipulations. On his way home, August 6th, 1881, in the evening, the hernia slipped out beneath the truss-pad and soon became strangulated. Dr. Talbot saw him at midnight, two hours afterwards, and found him with cold, clammy skin; pulse 130. Hot applications and morphine were administered, and the next morning taxis was tried, but without success. During the day taxis was again tried under chloroform, but without avail. At 11 o'clock next day, August 8th, I saw the patient with Drs. Talbot, Lang and Collins. By the examination an enormous protrusion was seen, expanding the scrotum, being as large as the head of a child one year old. The skin was of a dark-red color, here and there with abrasions and blue contusions from the taxis. The whole protrusion was hard, almost solid, but with tympanitic percussion and not emphysematous, the pulse was still good and the patient anxious for operative help. Ether having been administered, a severe and prolonged attempt of taxis was made, lasting ¾ hour, but without any result at all. Herniotomy was therefore performed in the usual way, and the sac opened. A large coil of bowels, measuring about one foot in length, presented itself. Although extremely congested, the bowels were intact and were reposited after a great deal of trouble and delay, owing to a band at the internal opening of the canal, which escaped detection when the canal was incised. After the bowels had been disposed of, almost the whole omentum was discovered lying behind in the sac, and forming one roll, a foot long—firm, dark, and congested and adherent to the sac. The whole omentum was securely ligated with strong carbolized silk, cut off and the stump left in situ. The wound was well cleaned with carbolized water, a large drainage-tube introduced in the enormous sac, the wound sutured except at the lower angle, where the ligatures and the tube came out, and Lister's antiseptic bandage applied. The operation lasted about one hour. Contrary to our expectations he recovered without an unfavorable symptom, and the wound united by first intention. The bowels moved on the 4th day, and on the 9th he sat up and walked across the room. On the 15th day one of the ligatures came away, the other is still not loose. Twenty-two days after the operation he was able to go out. This case is of interest on account of the size of the protrusion, and of the amount of omentum cut off. The successful result of so formidable an operation shows that the operation was made in time, as far as the bowels were concerned. It is a well known fact that inguinal hernia, especially large ones, endure the strangulation for a longer time than femoral hernia, in which gangrene may appear inside of twenty-four hours. Still, I firmly believe, that life was saved by cutting off the whole congested and inflamed omentum, which without doubt would have produced diffuse peritonitis, if returned into the abdominal cavity.

